# Associations between sarcopenia and degenerative lumbar scoliosis in older women

**DOI:** 10.1186/s13013-017-0116-0

**Published:** 2017-03-16

**Authors:** Yawara Eguchi, Munetaka Suzuki, Hajime Yamanaka, Hiroshi Tamai, Tatsuya Kobayashi, Sumihisa Orita, Kazuyo Yamauchi, Miyako Suzuki, Kazuhide Inage, Kazuki Fujimoto, Hirohito Kanamoto, Koki Abe, Yasuchika Aoki, Tomoaki Toyone, Tomoyuki Ozawa, Kazuhisa Takahashi, Seiji Ohtori

**Affiliations:** 1 Department of Orthopaedic Surgery, Shimoshizu National Hospital, 934-5, Shikawatashi, Yotsukaido, Chiba 284-0003 Japan; 2 Department of Orthopaedic Surgery, Graduate School of Medicine, Chiba University, 1-8-1 Inohana, Chuo-ku, Chiba 260-8670 Japan; 3 Department of Orthopaedic Surgery, Eastern Chiba Medical Center, 3-6-2, Okayamadai, Togane, Chiba 283-8686 Japan; 4 Department of Orthopaedic Surgery, Showa University School of Medicine, 1-5-8 Hatanodai, Shinagawa-ku, Tokyo, 142-8555 Japan

**Keywords:** Adult spinal deformity, Sarcopenia, Skeletal muscle, Low back pain, Sagittal alignment

## Abstract

**Background:**

Age-related sarcopenia can cause various forms of physical disabilities. We investigated how sarcopenia affects degenerative lumbar scoliosis (DLS) and lumbar spinal canal stenosis (LSCS).

**Methods:**

Subjects comprised 40 elderly women (mean age 74 years) with spinal disease whose chief complaints were low back pain and lower limb pain. They included 15 cases of DLS (mean 74.8 years) and 25 cases of LSCS (mean age 72.9 years).

We performed whole-body dual-energy X-ray absorptiometry (DXA) to analyze body composition, including appendicular and trunk skeletal muscle mass index (SMI; lean mass (kg)/height (m)^2^) and bone mineral density (BMD). A diagnostic criterion for sarcopenia was an appendicular SMI <5.46. To check spinal alignment, lumbar scoliosis (LS), sagittal vertical axis (SVA), thoracic kyphosis (TK), lumbar lordosis (LL), pelvic tilt (PT), pelvic incidence (PI), sacral slope (SS), and vertebral rotational angle (VRA) were measured. Clinical symptoms were determined from the Japanese Orthopedic Association scores, low back pain visual analog scale, and Roland-Morris Disability Questionnaire (RDQ). Criteria for DLS were lumbar scoliosis >10° and a sagittal vertical axis (SVA) >50 mm. Sarcopenia prevalence, correlations between spinal alignment, BMD, and clinical symptoms with appendicular and trunk SMIs, and correlation between spinal alignment and clinical symptoms were investigated.

**Results:**

DLS cases had significantly lower body weight, BMI, lean mass arm, and total lean mass than LSCS cases. Sarcopenia prevalence rates were 4/25 cases (16%) in LSCS and 7/15 cases (46.6%) in DLS, revealing a high prevalence in DLS. Appendicular SMIs were DLS 5.61 and LSCS 6.13 (*p* < 0.05), and trunk SMIs were DLS 6.91 and LSCS 7.61 (*p* < 0.01) showing DLS to have significantly lower values than LSCS. Spinal alignment correlations revealed the appendicular SMI was negatively correlated with PT (*p* < 0.05) and the trunk SMI was found to have a significant negative correlation with SVA, PT, LS, and VRA (*p* < 0.05). The trunk SMI was found to have a significant positive correlation with BMD (*p* < 0.05). As for clinical symptoms, RDQ was negatively correlated with appendicular SMI and positively correlated with PT (*P* < 0.05).

**Conclusions:**

Sarcopenia complications were noted in 16% of LSCS patients and a much higher percentage, or 46.6%, of DLS patients. Appendicular and trunk SMIs were both lower in DLS, suggesting that sarcopenia may be involved in scoliosis. The appendicular skeletal muscle was related to posterior pelvic tilt, while the trunk muscle affected stooped posture, posterior pelvic tilt, lumbar scoliosis, and vertebral rotation. Decreases in trunk muscle mass were also associated with osteoporosis. Moreover, RDQ had a negative correlation with appendicular skeletal muscle mass and a positive correlation with PT, suggesting that sarcopenia may be associated with low back pain as a result of posterior pelvic tilt. Our research reveals for the first time how sarcopenia is involved in spinal deformations, suggesting decreases in pelvic/lumbar support structures such as trunk and appendicular muscle mass may be involved in the progression of spinal deformities and increased low back pain.

## Background

As our society continues to age, more patients develop kyphotic deformities that affect their daily activities. Takemitsu et al. [1] reported that patients suffer disruption of their ADL and low back pain as a result of posterior lumbar tilt. A broad range of associated issues can impact ADL including low back pain due to spinal deformation, back pain, and gait disorders accompanying trunk imbalance, gastroesophageal reflux disease, and esthetic and psychological complaints [1–6]. Various causes of degenerative lumbar scoliosis (DLS) have been reported, including sex, age, osteoprotic vertebral fractures, kyphosis due to deformity, and factors due to spinal surgery, but the disease mechanism is yet to be elucidated [1–6]. Trunk muscles play an important role in the spinal support structure, and paraspinal muscle degeneration has been reported to be related to spinal deformity. However, there are no reports on the relationship between trunk and appendicular skeletal muscle mass and spinal deformation.

Sarcopenia is a syndrome characterized by progressive and systemic loss of skeletal muscle mass and muscle strength. It is an at-risk state where a fall could easily lead to the patient becoming bedridden, and it can lead to major physical and economic losses in an aging society [7–9]. It is believed to be caused by inactivity, but this mechanism has not yet been completely elucidated. Sarcopenia causes decreases in back strength, and this is believed to be a factor in aggravating kyphosis, but there are no clear research results on how sarcopenia affects DLS.

In this study, we looked at how sarcopenia is associated with degenerative lumbar scoliosis (DLS) and lumbar spinal canal stenosis (LSCS) and at the relationship between spinal alignment and skeletal musculature.

## Methods

Subjects included 40 women with spinal disease and a chief complaint of low back pain or lower limb pain (mean 74.0 ± 1.0 years). There were 15 cases (mean 74.8 ± 1.3 years) of DLS and 25 cases (mean 72.9 ± 1.4 years) of LSCS. There were 3 patients with L5 foraminal stenosis but without central canal stenosis in the DLS group. There were no patients with lumbar scoliosis in the LSCS group. Five cases in the DLS group recieved corrective surgery, while all cases in the LSCS group underwent laminectomies Exclusion criteria included a history of multiple fractures of the thoracolumbar spine, spinal surgery or hip joint surgery, and neuromuscular disorders such as Parkinson’s disease. Criteria for DLS were lumbar scoliosis >10°, and a sagittal vertical axis (SVA) of >50 mm^2^.

Body composition was measured using whole-body dual-energy X-ray absorptiometry (DXA) (Hologic, QDR-DELPHIW scanner DPX-NT; Hologic, Waltham, MA, USA). This system provided the mass of lean soft tissue, fat, and bone mineral for both the whole body and specific regions such as the arms, legs, and trunk.

Appendicular skeletal muscle mass was calculated as the sum of skeletal muscle mass in the arms and legs, assuming that the mass of lean soft tissue is a skeletal muscle. Appendicular skeletal mass index (SMI) was determined as the sum of the arm and leg lean mass (kg)/height^2^ (m^2^). Sarcopenia among the women was defined as an appendicular SMI value of <5.46 kg/m^2^ based on normative data for sarcopenia in Japanese men and women [10]. Although the lean mass of the trunk contains the internal organs, relative trunk SMI was defined as the trunk lean mass (kg)/height^2^ (m^2^). Age, height, weight, body mass index (BMI), bone mineral density (BMD), lean mass arm, lean mass leg, lean mass trunk, appendicular lean mass, and total lean mass were recorded for all patients (Table 1).

**Table 1:** Patient characteristics

	DLS	LSCS	*p* value
Age (years)	74.8 ± 1.3	72.9 ± 1.4	0.326
Body weight (kg)	46.6 ± 1.1	53.1 ± 1.9	0.008
Height (m)	1.51 ± 0.01	1.49 ± 0.01	0.391
BMI (kg/m^2^)	20.3 ± 0.5	23.8 ± 0.6	0.0004
BMD	0.962 ± 0.033	0.949 ± 0.013	0.703
Lean mass arm (kg)	2.82 ± 0.11	3.34 ± 0.10	0.003
Lean mass leg (kg)	9.88 ± 0.20	10.58 ± 0.38	0.201
Lean mass trunk (kg)	1.58 ± 0.32	1.69 ± 0.45	0.053
Appendicular lean mass (kg)	12.71 ± 0.35	13.93 ± 0.47	0.079
Total lean mass (kg)	29.59 ± 1.98	34.27 ± 0.94	0.021

The frontal view of the entire spine and the lateral view including the hip joints were photographed in a standing position. Radiographic measurements were made of lumbar scoliosis (LS), sagittal vertical axis (SVA), thoracic kyphosis (TK), lumbar lordosis (LL), pelvic tilt (PT), pelvic incidence (PI), and sacral slope (SS). Vertebral rotational angle (VRA) was measured in the axial computed tomography (CT) plane. The LS was measured as the angle between the lower end plate of L1 and the lower end plate of L5 on frontal radiographs. The SVA was measured as the distance from the C7 plumb line to a perpendicular line drawn from the superior posterior end plate of the S1 vertebral body on lateral radiographs. The TK was measured from the upper end plate of T5 to the lower end plate of T12. The LL was measured from the lower end plate of T12 to the upper end plate of S1. The PT was measured as the angle between the vertical line and the line joining the hip axis to the center of the superior end plate of S1. The PI was measured as the angle subtended by a perpendicular line from the upper end plate of S1 and a line connecting the center of the femoral head to the center of the cephalad end plate of S1. The SS was measured as the angle between the superior end plate of S1 and a horizontal line. Vertebral rotational angle (VRA) was defined as the angle between longitudinal axis of the apical vertebra and the midsagittal axis of the sacral vertebra.

Clinical symptoms were evaluated using the visual analog scale (VAS) score for low back pain from 100 (extreme amount of pain) to 0 (no pain), the Japanese Orthopedic Association (JOA; 0–29 points) scoring system and the Roland-Morris Disability Questionnaire (RDQ; 0–24 point). The normal JOA score is 29 points, based on 3 subjective symptoms (9 points), 3 clinical signs including straight-leg raising (6 points), and 7 activities of daily living (14 points). The normal RDQ is zero points with the total number of items checked from a minimum of 0 to a maximum of 24.

Study items were sarcopenia prevalence in each group, correlations between spinal alignment, BMD, and clinical symptoms with appendicular and trunk SMIs, and correlation between spinal alignment and clinical symptoms.

### Statistical analysis

Statistical analyses were performed with Stat View software (version 5.0).

For each parameter, differences between both groups were evaluated by the unpaired *t* test.

Pearson correlation coefficients were calculated to determine the correlation between appendicular SMI or trunk SMI and spinal parameters or clinical symptoms. A threshold of *p* < 0.05 was considered significant.

## Results

Subject heights were DLS 1.51 ± 0.01 m and LSCS 1.49 ± 0.01 m (*p* = 0.391); body weight was DLS 46.6 ± 1.1 kg and LSCS 53.1 ± 1.9 kg (*p* < 0.01); BMI was DLS 20.3 ± 0.5 and LSCS 23.8 ± 0.6 (*p* < 0.001); BMD was DLS 0.962 ± 0.033 and LSCS 0.949 ± 0.013 (*p* = 0.703); lean mass arm was DLS 2.82 ± 0.11 kg and LSCS 3.34 ± 0.10 kg (*p* < 0.01); lean mass leg was DLS 9.88 ± 0.20 kg and LSCS 10.58 ± 0.38 kg (*p* = 0.201); lean mass trunk was DLS 1.58 ± 0.32 kg and LSCS 1.69 ± 0.45 kg (*p* = 0.53); appendicular lean mass was DLS 12.71 ± 0.35 kg and LSCS 13.93 ± 0.47 kg (*p* = 0.079); and total lean mass was DLS 29.59 ± 1.98 kg and LSCS 34.27 ± 0.94 kg (*p* < 0.05). DLS cases had significantly lower body weight, BMI, lean mass arm, and total lean mass than LSCS cases (Table 1).

Radiographical alignment in the DLS group revealed SVA 78.6 ± 7.3 mm, LS 29.9 ± 2.4°, TK 18.4 ± 3.8°, LL 26.2 ± 4.9°, PI 55.7 ± 3.5°, PT 32.3 ± 2.7°, and SS 25.6 ± 3.3°. In the LSCS group, SVA 32.2 ± 4.2 mm, LS 4.1 ± 0.8°, TK 24.5 ± 1.5°, LL 41.5 ± 2.5°, PI 49.0 ± 2.4°, PT 22.1 ± 1.2°, and SS 28.7 ± 2.0°.

**Fig. 1:**
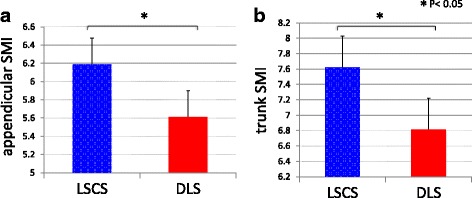
Appendicular and trunk SIMs in both groups. **a** Appendicular SIMs were ASD 5.61 ± 0.16 and LSCS 6.13 ± 0.15 (*p* < 0.05), and **b** trunk SIMs were DLS 6.91 ± 0.17 and LSCS 7.61 ± 0.15 (*p* < 0.01). DLS values were significantly lower than those of LSCS

Sarcopenia prevalence was DLS 7/15 cases (46.6%) and LSCS 4/25 cases (16%) with a high percentage of involvement in DLS cases. Appendicular SMIs were DLS 5.61 ± 0.16 and LSCS 6.13 ± 0.15 (*p* < 0.05); trunk SMI values were DLS 6.91 ± 0.17 and LSCS 7.61 ± 0.15 (*p* < 0.01) with DLS significantly lower than LSCS (Fig. 1). In this study, since there are more severe coronal deformity parameters (LS 29.9°) than sagittal balance parameter (SVA 78.6 mm) in DLS cases, we analyzed DLS cases into coronal scoliosis subgroups, high coronal scoliosis (HS) group (LS > 30°; average 36.8°), and low coronal scoliosis (LS) group (LS < 30°; average 23.0°). Appendicular SMI was 5.92 ± 0.30 in the HS group, versus 5.36 ± 0.07 in the LS group (*p* = 0.10); trunk SMI was 6.90 ± 0.30 in the HS group, versus 6.97 ± 0.21 in the LS group (*p* = 0.85). Differences were not found between appendicular or trunk SMI in the HS group and in LS group.

**Fig. 2:**
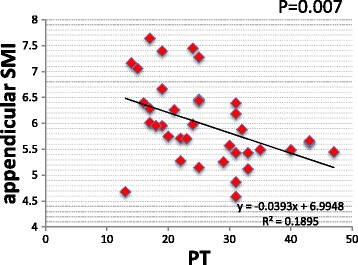
Correlation with appendicular SMI. A statistically significant negative correlation was noted between appendicular SMI and PT (*p* < 0.05)

**Fig. 3:**
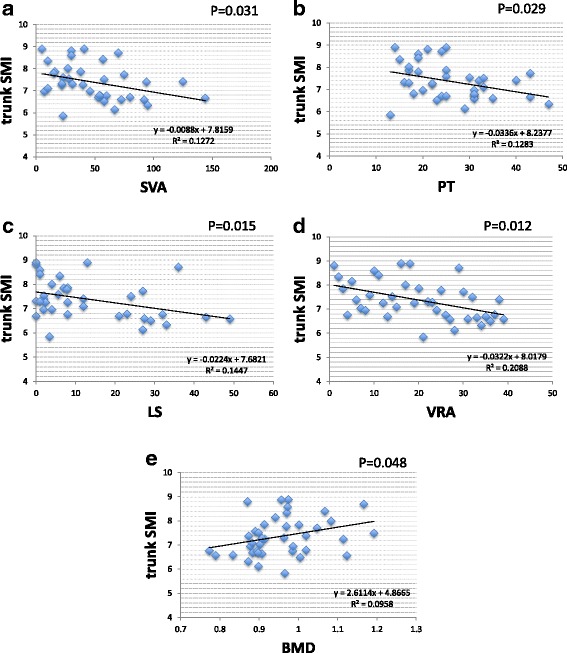
Correlation with trunk SMI. A statistically significant negative correlation was observed between trunk SMI and SVA (**a**), PT (**b**), LS (**c**), and VRA (**d**) (*p* < 0.05). A statistically significant positive correlation was observed between trunk SMI and BMD (**e**) (*p* < 0.05)

**Fig. 4:**
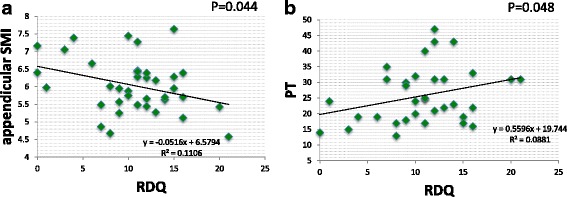
Correlation with a scale of clinical symptoms, RDQ. A statistically significant negative correlation was noted between appendicular SMI and RDQ (**a**) (*p* < 0.05). A statistically significant positive correlation was noted between PT and RDQ (**b**) (*p* < 0.05)

Correlations with spinal alignment revealed a significant negative correlation between appendicular SMI and PT (*p* < 0.05) (Fig. 2). Negative correlations between trunk SMI and SVA, PT, LS, and VRA were also statistically significant (*p* < 0.05) (Fig. 3a–d). Trunk SMI was found to have a significant positive correlation with BMD (*p* < 0.05) (Fig. 3e). As for clinical symptoms, there was a negative correlation (*p* < 0.05) between appendicular SMI and RDQ (Fig. 4a) and a positive correlation (*p* < 0.05) between PT and RDQ (Fig. 4b).

## Discussion

Reports have been published on research using MRI to assess paraspinal muscle in spinal deformities. Yagi et al. [11] reported that multifidus and iliopsoas muscle cross sections were smaller in spinal deformation, and that this correlated with sagittal alignment. A report found fatty degeneration of multifidus muscle on the concave side of degenerative scoliosis [12], while hyperplasia of the multifidus muscle and iliopsoas muscle has been reported regarding the convex side of degenerative scoliosis [13]. On the other hand, when Enomoto et al. [14] took surface electromyograms of paravertebral muscle activity, they found that compared to lumbar spinal canal stenosis (LSCS), patients with degenerative lumbar scoliosis (DLS) had high paravertebral muscle activity. Yagi et al. [11] measured appendicular skeletal muscle mass in patients with DLS and LSCS by DXA and reported that there was no significant difference between the two groups. However, postoperative measurements were only taken for appendicular weight, and height-corrected SMI values were not considered. Muscle assessment in adult spinal deformity had previously been limited to localized evaluation of appendicular and trunk muscle mass. How these might relate to sarcopenia has never been investigated until this time.

Sarcopenia is defined as the age-associated loss of skeletal muscle mass and function with a risk of adverse outcomes such as physical disability and poor quality of life [7, 8]. Sarcopenia is very common in older individuals, with a reported prevalence in 60- to 70-year-olds of 5–13% [9].

In a report on sarcopenia and spinal diseases, Miyakoshi et al. [15] reported 20% of Japanese patients with osteoporosis suffer sarcopenia complications while only 10% of healthy individuals have sarcopenia. In our study, trunk SMI was found to have a significant positive correlation with BMD, suggesting that decreases in trunk muscle mass were associated with osteoporosis. Another study found that patients with low back pain have a statistically significant decrease in lower appendicular muscle mass [16]. However, no studies have clearly defined the relationship between sarcopenia and spinal deformity.

With regard to spinal alignment which adversely affects QOL, Takemitsu et al. [1] reported that 95% of patients with lumbar degenerative kyphosis suffer low back pain with severe disruption of their ADL and raised these issues regarding kyphosis. Glassmann et al. [3] found that those cases with large SVAs, where the C7 plumb line shows anterior displacement, suffer the greatest disruption of QOL and stressed the importance of sagittal alignment. Lafage et al. [4] have associated posterior pelvic tilt and stooping posture to poor QOL and so consider PT and SVA to be vital factors. Schwab et al. [5] chose radiographical parameters PI-LL <10°, PT < 20°, and SVA < 50 mm as the thresholds for correction and mentioned the importance of a good sagittal plane balance.

**Fig. 5:**
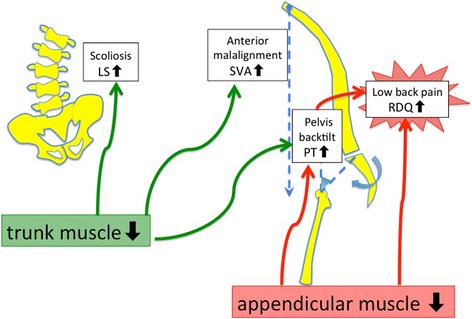
Skeletal muscle mass and relationship with spinal alignment and lumbar pain. Findings suggested loss of skeletal muscle is related to posterior pelvic tilt (PT increase) and low back pain (RDQ increase). Loss of trunk muscle may be related to anterior tilt (SVA increase), posterior pelvic tilt (PT increase), lumbar scoliosis (LS increase), and vertebral rotation (VRA increase)

In our research, sarcopenia complications were found in 16% of LSCS, and nearly half, or 46.6% of DLS. Appendicular SMI and trunk SMI were both reduced in DLS, suggesting that sarcopenia may be involved in scoliosis. In particular, lean mass arm and total lean mass were markedly reduced in DLS compared with LSCS. Differences might be not found in lean mass leg between DLS and LSCS due to disuse atrophy from intermittent claudication in LSCS. In the future, results should be compared to a healthy volunteer without back problems. Moreover, appendicular skeletal muscle mass was negatively correlated with PT, while trunk muscle mass showed negative correlations with SVA, PT, LS, and VRA. Appendicular skeletal muscle was associated with posterior pelvic tilt, while trunk muscle mass was associated with stooped posture, posterior pelvic tilt, lumbar scoliosis, and vertebral rotation. In addition, RDQ had a negative correlation with appendicular skeletal muscle mass and a positive correlation with PT, suggesting a relationship between sarcopenia and low back pain as a result of posterior pelvic tilt (Fig. 5). Our results do not differ from those of published reports and confirm that sagittal plane alignment PT and SVA are important factors that affect QOL. Decreases in trunk muscle and appendicular muscle mass which form the pelvic/lumbar stabilization structure may be one of the causes of spinal deformation and low back pain.

Our study has several limitations. (1) The first is that only a small number of subjects were investigated, requiring confirmation of our findings in a larger population.

(2) DXA cannot measure individual spinal muscles such as paravertebral muscle and psoas. The trunk SMI defined in this study includes the internal organs so it is not an accurate measure of actual trunk muscle volume but merely a relative evaluation. However, trunk muscle accounts for approximately 15% of the lumboabdominal region and is second only to the 30% representing the femoral muscles, and so it cannot be ignored in terms of assessing whole-body skeletal muscle mass [17]. A new device has recently been introduced to evaluate the total and regional body composition—bioelectrical impedance analyzer (BIA). BIA estimates body composition using the difference of conductivity of the various tissues due to the difference of their biological characteristics. High agreement between DXA and BIA was high for lean mass trunk (95%IC 0.82) [18]. In the future, results should be compared to measure the trunk muscle with BIA and MRI evaluations. (3) The study is a cross-sectional analysis, not a longitudinal one. (4) This study was only compared to patient with spinal stenosis but not compared to a normal population without back problems and not compared to younger populations.

(5) We did not study postoperative spinal alignment, but multifidus muscular atrophy has been implicated in proximal junctional kyphosis (PJK) [19] after orthomorphic surgery [11] and should be investigated further in the future.

## Conclusions

We investigated how sarcopenia affected degenerative lumbar scoliosis (DLS) and lumbar spinal canal stenosis (LSCS) in elderly women. Corrected appendicular muscle mass and corrected trunk muscle mass were determined using DXA. Sarcopenia was noted in 16% of LSCS and a much higher 46.6% of patients with DLS. Both appendicular and trunk skeletal muscle mass was lower in the DLS group, suggesting sarcopenia may be involved in causing spinal deformities. Decreases in appendicular skeletal muscle mass were associated with posterior pelvic tilt and low back pain, while decreases in trunk muscle mass were associated with stooping posture, posterior pelvic tilt, lumbar scoliosis, vertebral rotation, and osteoporosis. Low back pain was associated with decreased appendicular skeletal muscle mass and posterior pelvic tilt.

Loss of trunk and appendicular muscle, which form the truncal stabilization structure, is thought to be one of the causes of progressive deformation of the spine and low back pain.

## Abbreviations

BMD: Bone mineral density
DLS: Degenerative lumbar scoliosis
DXA: Dual-energy X-ray absorptiometry
LL: Lumbar lordosis
LS: Lumbar scoliosis
LSCS: Lumbar spinal canal stenosis
PI: Pelvic incidence
PT: Pelvic tilt
RDQ: Roland-Morris Disability Questionnaire
SMI: Skeletal muscle mass index
SS: Sacral slope
SVA: Sagittal vertical axis
TK: Thoracic kyphosis
VAS: Visual analog scale
VRA: Vertebral rotational angle
